# A Comprehensive Review on Upper Tract Urothelial Carcinoma: An Update in 2023

**DOI:** 10.3390/cancers16091613

**Published:** 2024-04-23

**Authors:** Alireza Ghoreifi, Hooman Djaladat

**Affiliations:** Institute of Urology, University of Southern California, Los Angeles, CA 90089, USA

It is our pleasure to serve as the guest editors for the *Cancers* journal for this Special Issue, titled “Comprehensive Review on Upper Tract Urothelial Carcinoma: An Update in 2023”. This Special Issue comprises nine manuscripts authored by experts in the field, covering various aspects of the diagnosis and management of upper tract urothelial carcinoma (UTUC). 

UTUC is a relatively uncommon type of cancer, which shares similarities with urothelial bladder cancer. Nevertheless, significant differences exist between these two cancer types in terms of epidemiological, clinical, pathological, and biological features. In a review article, Lefort et al. extensively explored these differences and their clinical implications [1]. The key steps in the management of patients with UTUC include precise diagnosis and risk stratification. Tsikitas et al. presented the latest advancements in imaging for UTUC [2]. The authors reviewed the strengths and weaknesses of conventional imaging techniques, including CT urography and magnetic resonance imaging (MRI), as well as other promising modalities, such as contrast-enhanced ultrasound (CEUS) and positron emission tomography (PET). They also highlighted the role of artificial intelligence and multiomics in the classification and prognostication of UTUC. In another paper, Bitaraf et al. reviewed other diagnostic tools (i.e., urine cytology and ureteroscopy), as well as patient- and disease-related prognostic factors that affect the outcomes of patients with UTUC [3]. They emphasized the substantial role of preoperative risk stratification tools and nomograms, which have been developed to guide surgical management and perioperative systemic therapy in UTUC. A real-world study in this context was presented by Huang et al., who reviewed the outcomes of 476 patients with pT2N0M0 UTUC undergoing radical nephroureterectomy (RNU) or ureterectomy [4]. They found that age >60 years, previous bladder cancer history, ureteral involvement, and positive surgical margins were independently associated with negative oncological outcomes.

The gold standard for the management of UTUC is RNU with bladder cuff excision. During the recent two decades, there has been a major shift from open RNU towards minimally invasive techniques. Franco et al. presented the latest evidence regarding surgical techniques and outcomes of minimally invasive RNU, focusing on robotic RNU [5]. The authors reviewed novel robotic techniques, including single-stage transperitoneal, retroperitoneal, and single-port RNUs. Another evolution in the management of UTUC has been kidney-sparing surgery, which emerged as the preferred option for select patients, particularly those with a low-risk disease. Ghoreifi et al. reviewed the outcomes of these techniques, including endoscopic ablation and segmental ureterectomy [6]. Several retrospective comparative studies have confirmed the feasibility and efficacy of kidney-sparing management approaches for UTUC, yet the only level I evidence so far in this setting is mitomycin gel therapy in low-risk patients.

Despite the technical advancements in the management of UTUC, oncologic outcomes are still not optimal. A multidisciplinary approach, incorporating perioperative intravesical and systemic therapy, has shown to improve these outcomes. Wang et al. comprehensively reviewed the medications, dosage, and timing of intravesical therapy for UTUC, and reported a reduced risk of intravesical recurrence and improved patient survival among those receiving this type of therapy [7]. In another study, Kolawa et al. reviewed the importance of perioperative systemic therapy in these patients and emphasized that neoadjuvant cisplatin-based therapy is preferred by clinicians over adjuvant therapy in high-risk patients, due to the potential decline in renal function following RNU [8]. The results of the ongoing trials have the potential to establish adjuvant immunotherapy as a potential new standard of care of UTUC.

All patients with UTUC require a close follow-up after a surgical intervention with curative intent. Klemm et al. presented surveillance protocols following definitive therapy for UTUC [9]. The surveillance modalities included urine cytology, cystoscopy, and CT/MR urography, and ureteroscopy (in kidney-sparing surgeries), with intervals varying according to risk stratification and the surgical approach used.

The management of UTUC has seen notable advancements in the recent decade. Nonetheless, this area is undergoing rapid evolution, and future studies will provide insights into the optimal approach for managing these patients.
